# Supplementary material for: XBP1 expression in pancreatic islet cells is associated with poor glycaemic control especially in young non-obese onset diabetes across ancestries
Source: Commun Med (Lond). 2025 Sep 24;5:396. doi: 10.1038/s43856-025-01076-2 (PMC12460670; doi:10.1038/s43856-025-01076-2)
Supplement: Supplementary file 3 — Description of Additional Supplementary files [file 43856_2025_1076_MOESM3_ESM.pdf]

Description of Additional Supplementary files

File name: Supplementary Data 1

Description: Supplementary Data used to make Figure 1

File name: Supplementary Data 2

Description: Supplementary Data used to make Figure 3
